# Adherence to the Mediterranean Diet in Relation to Gastric Cancer in Afghanistan

**DOI:** 10.3389/fnut.2022.830646

**Published:** 2022-03-28

**Authors:** Freshta Amiry, Seyed Mohammad Mousavi, Ahmad Mujtaba Barekzai, Ahmad Esmaillzadeh

**Affiliations:** ^1^Department of Community Nutrition, School of Nutritional Sciences and Dietetics, Tehran University of Medical Sciences, Tehran, Iran; ^2^Department of Public Health Management, School of Public Health, Kabul University of Medical Sciences, Kabul, Afghanistan; ^3^Obesity and Eating Habits Research Center, Endocrinology and Metabolism Molecular-Cellular Sciences Institute, Tehran University of Medical Sciences, Tehran, Iran; ^4^Food Security Research Center, Department of Community Nutrition, School of Nutrition and Food Science, Isfahan University of Medical Sciences, Isfahan, Iran

**Keywords:** diet, gastric cancer, Mediterranean diet (MD), case-control, Afghanistan

## Abstract

**Background:**

Despite the adherence to the Mediterranean dietary pattern (MD) being associated with a reduced risk of several cancers, there is no report about the highly prevalent diet-disease associations in Afghanistan, particularly about gastric cancer (GC).

**Objective:**

The aim of this study was to investigate the association between MD and GC in Afghanistan.

**Methods:**

This hospital-based case-control study was carried out on a total number of 270 subjects (90 cases and 180 controls) aged between 20 and 75 years. Using the convenience-sampling method, cases and controls were selected. Cases were patients with GC whose condition was pathologically confirmed. The controls were apparently healthy people who were matched with cases in terms of age (±5 years) and sex. Assessment of dietary intake was done using a pre-tested food frequency questionnaire, designed specifically for Afghanistan. Adherence to the MD pattern was done based on the scores suggested in earlier studies.

**Results:**

Out of 270 studied subjects, 73% were men. We found that subjects in the highest tertile of MD score had 52% decreased odds of GC (OR: 0.48; 95% CI: 0.24–0.98, *P*-trend = 0.05) compared with those in the lowest tertile. After considering potential environmental factors, age, and sex, the observed association disappeared (OR: 0.43; 95% CI: 0.13–1.38, *P*-trend = 0.41). After further adjustment for BMI in the last model, we found that participants with the highest MD score were 83% less likely to have GC than those in the lowest tertile (OR: 0.17; 95% CI: 0.03–0.80, *P*-trend = 0.14).

**Conclusion:**

We found that greater adherence to MD might be associated with a lower odds of GC.

## Introduction

Gastric cancer (GC) is one of the most common malignancies in the world; however, its prevalence has steadily declined over the past 50 years (1). Despite this reduction, GC is still the fifth most common cancer and the third most prevalent cause of cancer-related mortality (2). GC is highly prevalent in developing countries (3). Based on the recent WHO report, GC is the second most common cancer among Afghan men and the fourth in Afghan women (4).

Several lifestyle-related factors have been known to affect the risk of GC (4, 5). Among dietary factors, earlier studies have mostly focused on individual nutrients, while evaluating dietary patterns might be a more informative approach to clarify the link between nutrition and cancer (6). Mediterranean dietary pattern (MD) is characterized by high consumption of fruits, vegetables, whole grains, legumes, fish and seafood, nuts and seeds, olive oil as the main fat source, moderate alcohol consumption (particularly red wine), relatively low consumption of dairy products (mainly cheese and yogurt), and red and processed meat (7, 8). A recent systematic review and meta−analysis of observational studies provided evidence that greater adherence to the MD is associated with a lower risk of overall cancer-related deaths as well as lower incidence of colorectal, breast, prostate, liver, head and neck, and GC (9).

Most studies on the link between dietary factors and GC are limited to developed nations as limited information is available in developing countries, especially in Afghanistan. Given the particular nature of dietary intakes in Afghanistan along with the high prevalence of GC in this country (10, 11), it seems that investigating diet–GC relations in this country might move the field forward by providing additional information in this regard. Therefore, this study aimed to examine the association between adherence to the Mediterranean diet and the risk of GC in Afghanistan.

## Materials and Methods

### Study Participants

This hospital-based case-control study was carried out on a total number of 270 subjects aged between 20 and 75 years in Kabul, Afghanistan, in 2019. The sample size was calculated based on the formula for case–control studies (12). We hypothesized that almost 50% of Afghan adults are consuming unhealthy dietary patterns (high in fat and carbohydrate), so we assumed that such a dietary pattern might provide a 2.5 times greater risk of GC (13–15). Considering the type I error of 5%, study power of 80% (β = 0.2), and the ratio of cases to controls as 2, we reached 90 cases and 180 controls. A total of 90 new cases with GC who were pathologically diagnosed with cancer within the last month and 180 healthy controls who were matched with cases in terms of age (±5) and sex were selected. We excluded participants with a history of pathologically confirmed cancer (except for GC) or other types of cancer. Those with any type of other confirmed cancers (except GC) were excluded from the study. The study participants were matched in terms of age (±5 years) and sex. All study participants signed an informed written consent form before the initiation of data collection. The study was ethically approved by the medical bioethics committee of TUMS (Code: IR.TUMS.MEDICINE.REC.1399.184).

### Assessment of Dietary Intakes

Participants’ usual dietary intakes during the past year were examined by a pre-tested Willett-format food frequency questionnaire (FFQ), that was designed specifically for the Afghan population. The questionnaire included 103 food items, with standard portion sizes according to Afghan common dietary intakes. The FFQ was completed through face-to-face interviews by a trained interviewer in the presence of individuals who were involved in the preparation and cooking of foods. All reported consumption frequencies were converted to grams per day by using household measures. Daily intakes of energy and nutrients were calculated for each person by using the USDA food consumption database. Almost all foods included in the questionnaire were available in the database, whereas for those foods not available in the database, similar ones were considered. Similar foods were chosen based on similarities in macronutrients (proteins, carbohydrate, and fats). For example, we put the information for meat for Pacha (leg meat).

### Adherence to the Mediterranean Diet

We calculated MD score based on nine components [fruits, vegetables, fish, legumes, nuts, whole grains, the ratio of monounsaturated fatty acids (MUFAs) to saturated fatty acids (SFAs), meats (red meat, poultry, and proceed meats), and dairy products] as mentioned in earlier studies (16, 17). Participants received a score of 1 if they were above median intakes of fruits, fish, vegetables, legumes, nuts, whole grains, and the ratio of MUFA to SFA and below-median intakes of meats and dairy. They received 0 if their intake were more than the median of meats (red meat, poultry, and proceed meats) and dairy and less than the median intakes of fruits, fish, vegetables, legumes, nuts, whole grains, and the ratio of MUFA to SFA. The overall Mediterranean diet score was calculated by summing up the scores of each component. The range of MD scores was 0–9, with a higher score representing greater adherence to the MD.

#### Ascertainment of Gastric Cancer

The diagnosis of GC was confirmed based on the pathological test using the third edition of the International Classification of Diseases for Oncology (ICDO-3) and morphology codes 8490/3 (18, 19). We only enrolled GC patients who had been diagnosed for at most one year before the date of the interview.

### Assessment of Other Variables

A pre-tested questionnaire was used to collect data on demographic characteristics including age, BMI, physical activity, sex, marital status, education level, occupation, smoking status, alcohol consumption, tooth brushing (times per week), and kebab food consumption. By using an International Physical Activity Questionnaire (IPAQ), we assessed the physical activity of participants through the face-to-face interviews. The results of the IPAQ were expressed as Metabolic Equivalents per week (METs/week). A trained dietitian completed all measurements. Weight was measured to the nearest 0.1 kg using a digital scale (Seca, Hamburg, Germany) with minimal clothes and without shoes. By the use of a tape measure, we measured the height of participants in a standing position without wearing shoes to the nearest 0.1 cm. BMI was calculated as weight (kg) divided by height in (m^2^).

### Statistical Analysis

We categorized participants based on tertiles of the Mediterranean diet scores. The chi-square test was used to assess the distribution of categorical variables across tertiles of Mediterranean diet scores. We applied ANOVA for continuous variables to compare general characteristics of participants across tertiles of Mediterranean diet scores. For assessment of food and nutrients intakes of study participants across tertiles of MD score, we used analysis of covariance (ANCOVA) which was adjusted for age, sex, and total energy intake. To examine the association between Mediterranean diet scores and GC, we used binary logistic regression in three different models. First, we adjusted for age (continuous), and sex. Further adjustments were done for physical activity (continuous), marital status (married/not married), Kebab food consumption (yes/no), smoking (yes/no), tooth brushing (do not brush, brush), job status (former and worker, others), education (non-university graduate, university graduate), and alcohol use (yes/no) in the second model. To reach the obesity–independent relationships, further controlling was made for BMI in the final model. In all statistical analyses, the first tertile of Mediterranean diet score was used as the reference category. To obtain the overall trend of odds ratios across increasing tertiles of Mediterranean diet score, we considered tertiles as an ordinal variable in the logistic regression models. All statistical analyses were conducted using SPSS software version 25 (SPSS Inc., Chicago, IL, United States). *P*-values less than 0.05 were considered statistically significant.

## Results

Compared to controls, cases were more physically active (METs) (2.2 ± 0.9 vs. 1.6 ± 0.88, *P* ≤ 0.001) and more likely to be farmer and worker (36.7 vs. 15.6%, *P* ≤ 0.001), be current smokers (34.4 vs. 10.6%, *P* ≤ 0.001), and consume kebab food (78.9 vs. 30.6%, *P* ≤ 0.001). Also, cases had a lower mean of BMI (21.9 ± 3.72 vs. 25.2 ± 2.4, *P* ≤ 0.001), less likely to be university post-graduated (1.1 vs. 17.8%, *P* ≤ 0.001), and do tooth brushing (25.6 vs. 81.7%, *P* ≤ 0.001). There were no other significant differences in other variables between cases and controls.

General characteristics of study subjects across tertiles of MD score are shown in Table 1. Compared with those with the lowest score, participants who had higher MD scores were more likely to have post-graduate education and brush their teeth. Those in the top tertile of MD score were more likely to be married than those in the bottom tertile. There were no other significant differences in terms of mean age, BMI, and participants’ distribution in terms of categorical variables across the MD score.

**TABLE 1:** General characteristics and dietary habits of study participants.

	Controls (*n* = 180)	Cases (*n* = 90)	*P* **	Tertiles of MD score			
				
				1	2	3	*P* **
Age (years)	54.0 ± 11.7	55.1 ± 11.7	0.48	56 ± 12.2	54.6 ± 11.2	51.5 ± 11.3	0.58
BMI (kg/m^2^)	25.2 ± 2.4	21.9 ± 3.72	<0.001	23.9 ± 3.19	24.2 ± 3.38	24.2 ± 3.37	0.76
Physical activity (MET-h/week)	1.6 ± 0.88	2.2 ± 0.90	<0.001	2.97 ± 4.93	3.11 ± 6.01	4.74 ± 6.05	0.11
Male (%)	72.8	73.3	0.92	69.1	77.8	70.8	0.33
Married (%)	81.7	80	0.74	72.2	87	84.6	0.01
Job (farmer and worker, %)	15.6	36.7	<0.001	25.8	22.2	18.5	0.54
Post graduated education (%)	17.8	1.1	<0.001	4.1	13	23.1	0.001
Current smoker (%)	10.6	34.4	<0.001	16.5	17.6	23.1	0.101
Kebab food (%)	30.6	78.9	<0.001	30.9	21.3	16.9	0.09
Alcohol usage (%)	1.1	0.0	0.31	0.0	0.9	1.5	0.51
Tooth brushing (%)	81.7	25.6	<0.001	52.6	67.6	70.8	0.02

MD, Mediterranean diet, MET, metabolic equivalents, BMI, body mass index. *All values are mean ± SD unless indicated. **P-values were obtained from independent Student’s t-test or chi-square test, where appropriate.

Compared to controls, patients with GC had higher consumption of fish (6.13 ± 0.6 vs. 2.96 ± 0.42, *P* ≤ 0.001), meat products (59.65 ± 2.955 vs. 31.23 ± 2.08, *P* ≤ 0.001), total energy (3190.5 ± 109.4 vs. 2836.1 ± 77.37, *P* = 0.009), protein (91.4 ± .745 vs. 87.53 ± 0.524, *P* ≤ 0.001), MUFA (17.03 ± 0.51 vs. 15.81 ± .359, *P* = 0.053), and vitamins B1 (2.396 ± .28 vs. 2.321 ± 0.2, *P* = 0.033), B3 (29.89 ± 0.294 vs. 25.85 ± 0.207, *P* ≤ 0.001) and the had lower intakes of fruit (45.8 ± 6.6 vs. 105.2 ± 4.6, *P* ≤ 0.001), vegetables (412.4 ± 16.23 vs. 524.8 ± 11.42, *P* ≤ 0.001), nuts (4.698 ± 1.123 vs. 10.08 ± .79, *P* ≤ 0.001), legumes (73.14 ± 4.41 vs. 95.91 ± 3.107, *P* ≤ 0.001), dairy products (62.57 ± 9.413 vs. 172.4 ± 6.625, *P* ≤ 0.001), PUFA (25.12 ± .68 vs. 26.72 ± 0.479, *P* = 0.014), dietary fiber (17.62 ± 0.41 vs. 20.54 ± 0.288, *P* ≤ 0.001), vitamin B2 (0.993 ± .04 vs. 1.301 ± 0.028 *P* ≤ 0.001), B6 (1.221 ± 0.035 vs. 1.323 ± 0.025, *P* = 0.02), B9 (249.2 ± 7.504 vs. 286.8 ± 5.282, *P* ≤ 0.001), B12 (1.541 ± 0.074 vs. 1.716 ± 0.052, *P* = 0.017), calcium (575.8 ± 17.64 vs. 796.8 ± 12.42, *P* ≤ 0.001), and magnesium (200.9 ± 5.307 vs. 227.3 ± 3.735, *P* ≤ 0.001). Food and nutrients intakes of study participants across tertiles of MD score are presented in Table 2. Individuals in the highest tertile of MD score had higher consumption of fruit, vegetables, fish, legumes, nuts, total energy, total fat, dietary fiber, folate, magnesium and lower intakes of whole grain, carbohydrates, and niacin compared to those in the lowest tertile.

**TABLE 2:** Food and nutrients intakes of study participants.

	Controls (*n* = 180)	Cases (*n* = 90)	*P* **	Tertiles of MD score			*P* **
				
				1	2	3	
				
	Mean ± SE	Mean ± SE		Mean ± SE	Mean ± SE	Mean ± SE	
**Food groups**							
Fruits (g/d)	105.2 ± 4.6	45.8 ± 6.6	< 0.001	53.99 ± 6.726	90.1 ± 6.15	124.7 ± 8.256	< 0.001
Vegetables (g/day)	524.8 ± 11.42	412.4 ± 16.23	< 0.001	411.9 ± 15.97	510 ± 14.63	562.1 ± 19.61	< 0.001
Whole grains (g/day)	331.9 ± 13.88	365.1 ± 19.72	0.172	397.3 ± 19.2	317.6 ± 17.58	304 ± 0.2357	0.003
Fish (g/d)	2.96 ± 0.42	6.13 ± 0.6	< 0.001	2.9 ± 0.61	4.474 ± 0.562	4.936 ± 0.753	0.079
Legumes (g/day)	95.91 ± 3.107	73.14 ± 4.41	< 0.001	67.51 ± 4.196	93.25 ± 3.843	111.1 ± 5.151	< 0.001
Nuts (g/d)	10.08 ± 0.79	4.698 ± 1.123	< 0.001	4.873 ± 1.11	8.899 ± 1.016	12.37 ± 1.362	< 0.001
Dairy products (g/day)	172.4 ± 6.625	62.57 ± 9.413	< 0.001	137.1 ± 10.79	141.2 ± 9.883	124.9 ± 13.24	0.609
Total meats (g/day)	45.8 ± 2.53	80.0 ± 3.60	< 0.001	59.11 ± 3.94	58.77 ± 3.61	51.77 ± 4.84	0.44
Red meat	24.3 ± 1.62	43.6 ± 2.30	< 0.001	32.7 ± 2.46	32.3 ± 2.25	25.2 ± 3.01	0.11
Poultry	21.4 ± 1.58	36.3 ± 2.25	< 0.001	26.3 ± 2.35	26.4 ± 2.16	26.5 ± 2.89	0.99
**Nutrients**							
Energy (kcal/day)	2836.1 ± 77.37	3190.5 ± 109.4	0.009	2537.1 ± 100.9	3004.1 ± 95.30	3493.8 ± 123.4	< 0.001
Carbohydrates (g/day)	458.7 ± 3.665	416.07 ± 5.208	0.723	472.2 ± 5.079	453.2 ± 4.652	451 ± 6.235	0.010
Proteins (g/day)	87.53 ± 0.524	91.40 ± 0.745	< 0.001	89.32 ± 0.762	88.74 ± 0.698	88.22 ± 0.936	0.669
Total fats (g/day)	90.19 ± 1.616	86.54 ± 2.297	0.198	82.24 ± 2.229	92.05 ± 2.041	93.91 ± 2.736	0.001
Cholesterol (mg/day)	166.9 ± 7.299	162.6 ± 10.37	0.736	153.9 ± 10.25	168.4 ± 9.387	177.8 ± 12.58	0.34
SFA (g/day)	19.63 ± 0.461	18.85 ± 0.656	0.331	18.92 ± 0.649	20.06 ± 0.594	18.91 ± 0.797	0.334
MUFA (g/day)	15.81 ± 0.359	17.03 ± 0.510	0.053	15.28 ± 0.505	16.81 ± 0.462	16.62 ± 0.62	0.073
PUFA (g/day)	26.72 ± 0.479	25.12 ± 0.68	0.014	23.65 ± 0.651	27.17 ± 0.596	28.34 ± 0.799	0.082
Dietary fiber (g/day)	20.54 ± 0.288	17.62 ± 0.41	< 0.001	17.22 ± 0.388	20.17 ± 0.355	22.08 ± 0.476	< 0.001
Thiamine (mg/day)	2.321 ± 0.2	2.396 ± 0.28	0.033	2.412 ± 0.28	2.311 ± 0.025	2.305 ± 0.034	0.015
Riboflavin (mg/day)	1.301 ± 0.028	0.993 ± 0.04	< 0.001	1.102 ± 0.42	1.232 ± 0.039	1.286 ± 0.052	0.17
Niacin (mg/day)	25.85 ± 0.207	29.89 ± 0.294	< 0.001	28.11 ± 0.346	27.03 ± 0.316	26.10 ± 0.424	0.002
Vitamin B6 (mg/day)	1.323 ± 0.025	1.221 ± 0.035	0.02	1.137 ± 0.034	1.349 ± 0.031	1.417 ± 0.041	0.107
Folate (mcg/day)	286.8 ± 5.282	249.2 ± 7.504	< 0.001	239.6 ± 7.168	284.2 ± 6.565	309.3 ± 8.799	< 0.001
Cobalamin (mcg/day)	1.716 ± 0.052	1.541 ± 0.074	0.017	1.665 ± 0.074	1.756 ± 0.68	1.665 ± 0.074	0.385
Calcium (mg/day)	796.8 ± 12.42	575.8 ± 17.64	< 0.001	715.1 ± 20.64	727.8 ± 18.9	727.5 ± 25.34	0.891
Magnesium (mg/day)	227.3 ± 3.735	200.9 ± 5.307	< 0.001	195.6 ± 5.132	227 ± 4.7	238.5 ± 6.3	< 0.001

MD, Mediterranean diet; SFA, saturated fatty acid; MUFA, monounsaturated fatty acid; PUFA, polyunsaturated fatty acid; Fe, iron; Mg, magnesium. **All values were adjusted for energy intake, age, and sex, except for dietary energy intake, which was only adjusted for age and sex using ANCOVA.

Furthermore, four levels of multivariable-adjusted ORs for GC across tertiles of MD score are shown in Table 3. In the crude model, subjects in the highest tertile of MD score had 52% decreased odds of GC (OR: 0.48; 95% CI: 0.24–0.98, *P*-trend = 0.05) compared with those in the lowest tertile. After adjustment for age and sex in the first model, this protective association became slightly attenuated (OR: 0.49; 95% CI: 0.24–1.01, *P*-trend = 0.06). After further adjustment for potential environmental confounders, there was no significant association between adherence to MD score and GC (OR: 0.43; 95% CI: 0.13–1.38, *P*-trend = 0.41). When we included BMI in the last model, we found that participants with the highest MD score were 83% less likely to have GC than those in the lowest tertile (OR: 0.17; 95% CI: 0.03–0.8, *P*-trend = 0.14).

**TABLE 3:** Odds ratios (ORs) and 95% CIs of gastric cancer according to tertiles of the Mediterranean dietary score.

	Tertiles of MD score			*P*-trend*
	
	1	2	3	
	
	OR	OR (95% CI)	OR (95% CI)	
Crude	1.00	0.88 (0.49–1.57)	0.48 (0.24–0.98)	0.05
Model 1	1.00	0.69 (0.38–1.27)	0.49 (0.24–1.01)	0.06
Model 2	1.00	1.96 (0.82–4.68)	0.43 (0.13–1.38)	0.41
Model 3	1.00	1.40 (0.64–3.05)	0.32 (0.11–0.91)	0.09
Model 4	1.00	1.82 (0.69–4.80)	0.17 (0.03–0.80)	0.14

*Binary logistic regression was used to obtain OR and 95% CI. The overall trend of OR across increasing tertiles was examined by considering the median score in each category as a continuous variable.

*Model 1: Adjusted for age (continuous), sex (male/female).*

*Model 2: Further adjustments were made for physical activity (continuous), Marriage status (married/not married), Kebab food (yes/no), smoking usage (yes/no), tooth brushing (do not brush, brush), job) former and worker, others), Education (non-university graduate, university graduate), alcohol usage (yes/no).*

*Model 3: Model 2 without adjustment for Kebab food (yes/no).*

*Model 4: Additionally, adjusted for BMI (categorical).*

## Discussion

In this case–control study, we found that individuals with the greatest adherence to the Mediterranean diet had lower odds of GC than those with the lowest adherence. To the best of our knowledge, this is the First study in Afghanistan that examined the association between MD patterns and GC.

Gastric cancer is the third leading cause of cancer-related death globally. According to the World Cancer Research Fund, the incidence of stomach cancer was 6.1% worldwide in 2018 (2, 20). MD is linked to a lower risk of different chronic conditions including overall cancer incidence (21). In the current study, we found that participants with the highest adherence of MD score were 83% less likely to have GC than those with the lowest adherence. In line with our findings, an updated systematic review and meta-analysis on adherence to Mediterranean diet and risk of different cancers indicated that higher adherence to the MD was related to a lower risk of cancer mortality in the general population, and all-cause mortality among cancer survivors as well as colorectal, head and neck, respiratory, gastric, liver and bladder cancer risks (22). The observed associations are mainly explained by higher intakes of fruits, vegetables, and whole grains (23, 24). An earlier cohort study in the Netherlands showed that higher adherence to MD pattern was associated with reduced risks of esophageal and GC (24). In another cohort study that included 485,044 subjects (144,577 men) aged 35–70 years old from 10 European countries, after 8.9 years of follow-up (HR: 0.67; 95% CI: 0.47–0.94), higher adherence to MD was associated with a significant lower risk of incident GC (13). Higher scores of MD were also associated with a significantly lower risk of overall cancer mortality (by approximately 13%) as well as the incidence of GC (by 27%) in a meta-analysis (23). In addition, findings of a hospital-based case-control study in Iran demonstrated that a high-quality diet, that assessed by Healthy Eating Index-2010 and Mediterranean Style Dietary Pattern, was inversely associated with the risk of colorectal cancer (25). Similar findings were also reported from Italy (26). Overall, adherence to MD pattern seems to have beneficial effects on human health in particular against the incidence of GC. As this dietary pattern is more usual in Mediterranean countries, conducting studies in other countries like developing countries such as Afghanistan might be a very valuable step for moving the nutrition field forward in this regard.

With regard to the mechanisms through which the Mediterranean diet might affect the risk of GC, there are several foods in the context of this dietary pattern that can help reduce the risk of cancer. Several mechanisms include reducing the growth of tumor cells (e.g., fish consumption), increasing the chemo-protective effects (i.e., olive oil), and inhibiting tumor development (i.e., dairy products), anti-oxidative and anti-inflammatory effects (fruit, vegetables, and olive oil consumption) (27) might be involved in this process. In addition, antioxidants from this dietary pattern can reduce oxidative DNA damages eliminating free radicals and taking part in many biological changes linked with all cancers (e.g., bioactivation of carcinogens, cell signaling, cell regulation circle, angiogenesis, and inflammation). In stomach neoplasia, a high intake of fresh fruit and vegetable in this dietary pattern may reduce the negative effects of *Helicobacter pylori* and its consequent damages (28). Red meat and its process methods have been reported to be associated with an increased risk of GC (29).

The strengths of our study are being the first study in Afghan adults as well as considering a wide range of confounders. Some potential limitations should also be taken into account while interpreting our findings. Given the case–control design of the study, having recall bias and selection bias is unavoidable. In addition, causality cannot be inferred based on our findings due to the case-control design of the study. Because of the use of FFQ to assess dietary intakes, misclassification of study participants might have occurred. We tried our best to consider lots of confounders in the study; however, residual confounding cannot be ignored. Although we determined the sample size based on the suggested formula for case–control studies, larger studies seem to be required to confirm our findings. Finally, we did not consider sodium intake as a confounding factor, even though previous studies showed that the Mediterranean diet patterns have a limited effect on restricting sodium intake (30).

In conclusion, in this hospital-based case-control study we found that higher adherence to MD might be associated with a lower odds of GC. Further studies are needed to confirm this association.

## Data Availability Statement

The raw data supporting the conclusions of this article will be made available by the authors, on reasonable request to the corresponding author.

## Ethics Statement

This study was ethically approved by medical bioethics committee of TUMS (Code: IR.TUMS.MEDICINE.REC. 1399.184). The patients/participants provided their written informed consent to participate in this study.

## Author Contributions

AE and SM were involved in the study’s conception, design, statistical analysis, and interpretation of the data. FA and AB were involved in data collection, data cleaning, statistical analysis, and manuscript drafting. AE supervised the study. All authors approved the final manuscript for submission.

## Conflict of Interest

The authors declare that the research was conducted in the absence of any commercial or financial relationships that could be construed as a potential conflict of interest.

## Publisher’s Note

All claims expressed in this article are solely those of the authors and do not necessarily represent those of their affiliated organizations, or those of the publisher, the editors and the reviewers. Any product that may be evaluated in this article, or claim that may be made by its manufacturer, is not guaranteed or endorsed by the publisher.
